# Patient activation and self-management in patients with coronary heart disease: the mediating role of eHealth literacy

**DOI:** 10.3389/fpubh.2026.1799712

**Published:** 2026-09-11

**Authors:** Juan Tan, Yiyun Huang, Han Fu, Miyao Wu, Yin Hu, Feilan Jiang, Pengpeng Gao, Zhoumin Shen

**Affiliations:** 1 Operating Room, Hunan Provincial People’s Hospital (the First Affiliated Hospital of Hunan Normal University), Changsha, Hunan, China; 2 School of Nursing, Hunan Normal University, Changsha, Hunan, China; 3 Department of Emergency 2, Hunan Provincial People’s Hospital (the First Affiliated Hospital of Hunan Normal University), Changsha, Hunan, China; 4 Respiratory ICU, Hunan Provincial People’s Hospital (the First Affiliated Hospital of Hunan Normal University), Changsha, Hunan, China; 5 ICU of Emergency Department 3, Hunan Provincial People’s Hospital (the First Affiliated Hospital of Hunan Normal University), Changsha, Hunan, China; 6 Price Office, Hunan Provincial People’s Hospital (the First Affiliated Hospital of Hunan Normal University), Changsha, Hunan, China

**Keywords:** cardiac rehabilitation, chronic disease management, coronary artery disease, patient engagement, self-care

## Abstract

**Objective:**

Although patient activation and eHealth literacy are known to influence self-management in chronic diseases, evidence on whether eHealth literacy mediates this relationship in patients with coronary heart disease (CHD) remains limited. This study explored the associations between patient activation, eHealth literacy, self-efficacy and self-management in patients with CHD and aimed to examine both the direct and indirect associations of patient activation on self-efficacy and self-management through eHealth literacy.

**Methods:**

A cross-sectional method was adopted and a convenience sample of 433 hospitalized patients with CHD was recruited. Data were collected using the Patient Activation Measure-13, the eHealth Literacy Scale and the Heart Health Self-Efficacy and Self-Management scale (which provides a composite outcome measure) and analyzed using Pearson correlation, stepwise regression and bootstrap methods.

**Results:**

The mean scores were 58.89 ± 18.87 for patient activation, 24.92 ± 8.94 for eHealth literacy and 60.70 ± 15.34 for the combined self-efficacy and self-management measure. Patient activation was moderately positively correlated with eHealth literacy (*r* = 0.51, *p* < 0.001) and the combined measure (*r* = 0.59, *p* < 0.001), and eHealth literacy showed a weak positive correlation with the combined measure (*r* = 0.37, *p* < 0.001). Patient activation was directly and positively associated with the combined measure (*β* = 0.533, *p* < 0.001), and eHealth literacy partially mediated this relationship (indirect effect = 0.043, 95% CI [0.002, 0.088], *p* = 0.043), accounting for 9% of the total effect. This relatively small indirect effect suggests a modest mediating role, indicating that the direct pathway from patient activation to self-efficacy and self-management is substantially more influential than the pathway through eHealth literacy in this population.

**Conclusion:**

Patient activation, eHealth literacy, self-efficacy and self-management in patients with CHD were at moderate or moderately low levels. Higher levels of patient activation and eHealth literacy are associated with better self-efficacy and self-management, a pattern that may underlie improved self-management.

## 1 Introduction

Coronary heart disease (CHD) is a chronic condition characterized by stenosis or occlusion of the coronary arteries due to atherosclerosis, resulting in myocardial ischemia, hypoxia or necrosis (1). It is a major cause of death and disability worldwide and the leading cause of death in China (2, 3). According to the ‘Report on Cardiovascular Health and Diseases in China 2024’, the prevalence of CHD among residents aged 18 and older in China was 758 per 100,000 in 2023, with approximately 11.39 million prevalent cases nationwide (4). The mortality rate for CHD was 135.08 per 100,000 in urban residents and 148.19 per 100,000 in rural residents, and cardiovascular disease accounted for nearly 50% of all deaths among Chinese residents. Coronary heart disease imposes a substantial burden on patients, families and society, contributing to elevated healthcare expenditures, reduced quality of life, and increased risk of recurrent hospitalization, and hindering global public health development (5). Approximately 75% of CHD events can be prevented by modifying behaviors and managing risk factors, such as physical inactivity, unhealthy diet, smoking and psychological distress (6). Therefore, patients with CHD require long-term, comprehensive management to maintain cardiac health and prevent recurrence (7). To successfully perform such management, two interrelated but distinct personal resources are essential: self-efficacy, defined as an individual’s belief in their capacity to execute the behaviors necessary to achieve desired health outcomes, and self-management, defined as the individual’s ability to manage the symptoms, treatment, physical and psychosocial consequences, and lifestyle changes inherent in living with a chronic condition (8). Accordingly, enhancing the combined self-efficacy and self-management measure in patients with CHD has become a key focus in both research and clinical practice.

Patient activation refers to a patient’s knowledge, skills confidence and ability to manage their own health and care (9). And operationally, it is measured by the Patient Activation Measure (PAM-13), a validated instrument assessing self-reported knowledge, skills, and confidence for self-management. Grounded in Patient Activation Theory, the concept is both latent and behavioral, reflecting how individuals self-manage their health, understand their role and engage as active partners in their care (10). Individuals with higher patient activation demonstrate greater health literacy, more extensive disease knowledge and stronger self-care behaviors (11). They are more likely to engage in healthy behaviors such as exercise and healthy eating, as well as preventive actions such as receiving immunizations, attending medical appointments and adhering to prescribed medications (12). In contrast, individuals with lower activation levels are more likely to be hospitalized, have poorer treatment adherence, incur higher healthcare costs and report worse care experiences (13). Among patients with cardiovascular disease, low patient activation has been associated with reduced physical activity, multiple chronic conditions, smoking, older age (>65 years), and lower quality of life. For CHD patients navigating complex, lifelong treatment regimens, this issue is particularly salient (14). Reduced patient activation is associated with increased health risks and more severe health challenges in patients with CHD. Previous studies (15, 16) have shown that patient activation in individuals with CHD is positively correlated with both self-efficacy and self-management. Therefore, enhancing patient activation is critical for improving health outcomes, strengthening self-efficacy and promoting self-management in this population, and interventions targeting activation should be strengthened.

eHealth literacy, operationally defined as the perceived ability to seek, find, understand, and appraise health information from electronic sources and apply the knowledge gained to address a health problem (17), is measured by the eHealth Literacy Scale (eHEALS) It includes six domains: traditional literacy, health literacy, information literacy, scientific literacy, media literacy and computer literacy (18). This construct reflects patients’ comprehensive ability to use the internet to access health information. The concept has been extended to encompass digital health literacy, which refers to the ability to engage with digital technologies—including mobile health applications, wearable devices, telehealth platforms, and artificial intelligence (AI)-assisted patient education tools—in effective, safe and beneficial ways to achieve health goals. With the rapid development of information technology, the internet has become a key resource for disease prevention, treatment and management. It overcomes the limitations of traditional print media in disseminating health information, allowing the public to access health knowledge more conveniently and improving the effectiveness of health education and management for patients with chronic diseases. eHealth literacy is positively associated with multiple aspects of health promotion and care, influencing the acquisition, evaluation and application of health knowledge (19). Research shows that individuals’ perceived ability to use online health information affects their health behaviors and self-management, and limited skills may lead to adverse outcomes (20). However, whether eHealth literacy mediates the relationship between patient activation and self-efficacy and self-management in patients with CHD remains unexplored.

Previous studies (19–21) have provided preliminary evidence regarding the relationships among patient activation, eHealth literacy, self-efficacy, and self-management, yet notable gaps remain. Research has demonstrated that patient activation is positively correlated with eHealth literacy in older patients with chronic diseases (16), and that eHealth literacy serves as a mediator between social support and self-management in community-dwelling older adults with chronic non-communicable diseases (18). However, these studies have largely focused on heterogeneous chronic disease populations rather than CHD patients specifically. Other research has investigated the mediating role of self-efficacy in the relationship between health literacy and self-management behaviors among young and middle-aged CHD patients after percutaneous coronary intervention but did not incorporate digital health literacy as a psychobehavioral mediator. A recent study found positive correlations between eHealth literacy, compliance behavior, and quality of life in middle-aged and older CHD patients, suggesting that enhancing eHealth literacy can improve self-management and treatment adherence; however, this study examined only direct associations without testing mediation pathways. Consequently, the specific mechanism through which patient activation may influence self-efficacy and self-management via eHealth literacy in the CHD population has not been empirically examined.

Building on Social Cognitive Theory, which posits that personal factors (including self-efficacy), environmental factors, and behavior interact in a triadic reciprocal causation model, this study proposes a mediation framework. Within this framework, patient activation—representing an individual’s knowledge, skills, and confidence—can be understood as a personal determinant that drives patients to proactively seek, evaluate, and apply digital health information (i.e., eHealth literacy). Enhanced eHealth literacy, in turn, strengthens self-efficacy (a key personal factor) and promotes self-management behaviors. Specifically, more activated patients are more likely to actively navigate online health resources, critically evaluate the credibility of digital health information, and apply the acquired knowledge to daily disease management, thereby strengthening their self-management competencies and self-efficacy. This mediation pathway is consistent with the SCT proposition that behavioral engagement (information seeking and utilization) is facilitated by personal agency (patient activation) and reciprocally reinforces self-efficacy beliefs.

However, the potential benefits of eHealth literacy and digital health interventions in CHD self-management must be considered alongside concerns regarding the digital divide. Older adults, individuals with lower socioeconomic status, and those with limited educational attainment often face disproportionate barriers to accessing and effectively using digital health technologies. Among cardiovascular disease patients, age-stratified analyses have revealed that adults aged ≥65 years may require enhanced access and digital health literacy support to benefit from patient portals and telehealth services. These disparities in digital access and literacy may exacerbate existing health inequalities in CHD outcomes, underscoring the importance of understanding the role of eHealth literacy as a potential mediator and a target for equitable intervention.

Recent developments in digital health have further underscored the importance of eHealth literacy in cardiovascular care. Telehealth cardiac rehabilitation interventions have been shown to improve functional capacity, physical activity, and treatment adherence in patients with cardiovascular disease, with remotely monitored programs demonstrating comparable efficacy to traditional center-based rehabilitation (2). Mobile health applications and AI-assisted patient education tools have demonstrated potential in promoting lifestyle modification and medication adherence among CHD patients. However, the effectiveness of these digital interventions is contingent upon patients’ eHealth literacy, as limited digital skills may hinder their ability to benefit from such technologies.

Therefore, this study examines eHealth literacy as a mediating variable to determine whether it mediates the relationship between patient activation and “self-efficacy and self-management” in patients with CHD. By elucidating this mediation mechanism, the findings are expected to inform the development of eHealth literacy-based self-management interventions tailored to the CHD population, ultimately contributing to more effective and equitable chronic disease management strategies that integrate digital health competencies into patient activation enhancement programs.

## 2 Materials and methods

### 2.1 Participants

This cross-sectional study used convenience sampling to recruit hospitalized patients with CHD from the Department of Cardiology at Hunan Provincial People’s Hospital between May and June 2025. During the study period, all patients admitted to the department who met the eligibility criteria were consecutively invited to participate. No additional selection procedures were applied beyond the inclusion and exclusion criteria. Inclusion criteria were ① a diagnosis of CHD according to the guidelines of the American College of Cardiology and the American Heart Association (21), with a disease course of ≥3 months; and ② an age of ≥18 years. Exclusion criteria were ① the presence of severe mental illness, cognitive impairment or communication barriers that prevented completion of the questionnaire; and ② severe complications, such as heart failure. All participants provided written informed consent after being fully informed. Questionnaires were then distributed to the participants for completion.

Sample size was determined using two approaches. First, an *a priori* power analysis with G*Power 3.1.9.7 for the simple mediation model, assuming a small-to-medium effect size (*f*^2^ = 0.10), α = 0.05, and power = 0.95, indicated a minimum sample of 100 participants. Second, to accommodate the exploratory nature of the variable selection and guard against invalid responses, Kendall’s principle (22) was consulted, which recommended 182–364 participants based on the 29 variables and a 20% anticipated invalid response rate. Given that the primary objective is mediation analysis (investigating the statistical association between relevant factors and study outcomes), we further strengthened the methodological rigor by conducting an *a priori* Monte Carlo power analysis specifically for the indirect effect (9). To account for potential missing or invalid data, 447 questionnaires were collected, of which 433 were valid. This final sample size of 433 was confirmed by the Monte Carlo simulation to provide >0.99 statistical power for detecting the indirect effect, which far exceeds the recommended 0.80 level. Furthermore, a post-hoc analysis specifically for the mediation model corroborated this finding, yielding a statistical power >0.99 and fully satisfying the requirements for detecting mediation effects.

To ensure model stability and control for potential covariate confounding effects, 447 questionnaires were collected. After excluding 14 invalid questionnaires (3 due to missing baseline data required for calculations, e.g., BMI, and 11 with more than 6 “Not applicable” responses on the Patient Activation Measure-13 [PAM-13]), the final sample included 433 participants. Post-hoc analysis confirmed that this sample yielded a statistical power >0.99, fully satisfying the requirements for detecting mediation effects. Specifically, a post-hoc power analysis for the indirect effect in the simple mediation model, conducted via Monte Carlo simulation with 5,000 replications, confirmed that the final sample (*n* = 433) provided adequate power (>0.99) to detect a small-to-medium indirect effect, supporting the robustness of the mediation findings.

### 2.2 Data collection

All questionnaires were administered in Chinese. The general information questionnaire was developed in Chinese by the research team. The remaining instruments (the PAM-13, the eHealth Literacy Scale [eHEALS] and the Heart Health Self-Efficacy and Self-Management [HH-SESM] Scale) were originally developed in English, and validated Chinese versions were used. Details are as follows. For all scales, missing item-level data were minimal (<2%). Complete-case analysis was applied to the eHEALS and HH-SESM scales, as no participant had more than one missing item on either instrument.

#### 2.2.1 General information questionnaire

A self-developed questionnaire was used to collect demographic and disease-related data. Demographic variables included gender, age, education, employment status, marital status and body mass index (BMI). Disease-related variables included family history of cardiovascular disease (CVD), CHD-related healthcare visits in the past year, CHD duration, cardiac functional class and coronary stent count.

#### 2.2.2 Patient activation measure-13

The PAM-13, revised from the original PAM-22 by Hibbard et al. (9), includes 13 items across 4 dimensions. Items are scored on a 0–4-point Likert scale: 0 = ‘Not applicable’, 1 = ‘Strongly disagree’, 2 = ‘Disagree’, 3 = ‘Agree’ and 4 = ‘Strongly agree’. Total raw scores range from 0 to 52. Following Brenk–Franz and Hibbard (23), only questionnaires with at least 7 completed items were included. The PAM demonstrates high internal consistency (Cronbach’s alpha > 0.80) (24). For missing data, adjusted raw scores were calculated by dividing the sum of completed item scores by the number of items answered and multiplying the result by 13. Raw scores were then converted to a 0–100-point scale using a specific conversion table, with higher scores indicating greater patient activation. Based on these scores, participants were categorised into 4 levels: Level 1 (≤47.0 points), Level 2 (47.1–55.1 points), Level 3 (55.2–67.0 points) and Level 4 (≥67.1 points). The Chinese version of the PAM-13 has demonstrated good reliability (Cronbach’s *α* = 0.82) in patients with chronic heart failure. In this study, Cronbach’s α was 0.86.

#### 2.2.3 eHealth literacy scale

The eHEALS, developed by Norman et al. (17), comprises 8 items across 3 dimensions: application of online health information and services (items 1–5), evaluation ability (items 6–7) and decision-making ability (item 8). Items are rated on a 5-point Likert scale (1 = ‘Strongly disagree’, 2 = ‘Somewhat disagree’, 3 = ‘Neutral/Undecided’, 4 = ‘Somewhat agree’ and 5 = ‘Strongly agree’). Total scores can range from 8 to 40, with higher scores indicating greater eHealth literacy.

The eHEALS is the most widely used instrument for measuring eHealth literacy globally and has been translated into numerous languages and validated across diverse populations (10, 25). The Chinese version demonstrated excellent internal consistency (Cronbach’s *α* = 0.92) among Chinese undergraduates and supports a three-factor structure (1, 4, 5). Robust reliability has also been reported in other populations, including American patients with chronic disease (McDonald’s *ω* = 0.94) (6), Dutch patients with rheumatism (*α* = 0.93) (8, 9), and Portuguese health science students (*α* = 0.87) (7). In this study, Cronbach’s α was 0.88, indicating excellent internal consistency and aligning with prior research.

#### 2.2.4 Heart health self-efficacy and self-management

The HH-SESM scale, developed by Mares et al. (26) in 2020, includes 12 items across 6 dimensions: physical activity (items 1–2), diet (items 3–6), medication (items 7–8), psychosocial (items 9–10), weight management (item 11) and smoking (item 12). A 4-point Likert scale is used, and each item contributes to both the self-efficacy and self-management subscales. Total scores range from 24 to 96 points, encompassing individual subscale scores of 12–48 points for both self-efficacy and self-management. Scores ≥36 points indicate high levels, 25–35 points indicate moderate levels and ≤24 points indicate low levels of self-efficacy or self-management.

The scale has been translated and validated across cultures. The Chinese version, translated and validated by Mi et al. (27) in 400 Chinese patients with CHD, using Brislin’s back-translation method, demonstrated acceptable reliability (Cronbach’s α = 0.773 for self-efficacy and 0.721 for self-management). More recently, a Turkish version was validated among 250 patients with chronic heart disease, reporting Cronbach’s α values of 0.79 for self-efficacy and 0.81 for self-management, further confirming the scale’s robust psychometric properties across different populations (28). In this study, Cronbach’s α was 0.83 for self-efficacy and 0.81 for self-management, indicating good internal consistency and aligning with previous validation studies.

### 2.3 Procedure

During the data collection phase, researchers entered the patients’ wards accompanied by the head nurse of the cardiovascular department or a designated ward nurse. The nurse’s role was strictly limited to making the initial introduction before leaving the room. They were not present during the questionnaire completion and had no access to the individual responses, thereby ensuring that nursing staff were not involved in the data collection process. Subsequently, researchers clearly informed all potential participants that their decision to participate or decline would have no impact on their routine treatment, nursing care, or discharge plans, and that they could withdraw from the study at any time without providing a reason. The researchers then explained the purpose of the study, provided clear instructions on how to complete the questionnaire, and highlighted key precautions. Participants completed the questionnaires independently based on their actual circumstances, with no intervention from the researchers throughout the process. Upon completion, the researchers collected the questionnaires on the spot and conducted an immediate review. If any ambiguous or unclear responses were identified, the researchers directly verified them with the patients to ensure data accuracy.

### 2.4 Ethical

This study adhered to the ethical principles outlined in the Declaration of Helsinki. Ethical approval was obtained from the Ethics Committee of Hunan Provincial People’s Hospital (Ethics Review Number: No. (Health 17) of Lunshenke in 2021. Date: 2021.8.11).

### 2.5 Data analysis

To ensure data accuracy, information was entered and verified by two researchers. Statistical analyses were conducted using R software (R Foundation for Statistical Computing, Vienna, Austria). The following R packages were used: psych (for descriptive statistics and correlation analysis), lavaan (for confirmatory factor analysis), glmnet (for LASSO covariate selection), mediation (for bootstrap mediation analysis), and ggplot2 (for visualizations). Continuous variables are presented as mean and standard deviation, and categorical variables are summarized using frequencies and percentages. Pearson correlation analysis was used to evaluate the associations between variables. Mediation effects were assessed using the Baron–Kenny stepwise regression method, which involved building three models: a total effect model, a mediator path model and a direct effect model (29). Consistent with contemporary regression-based mediation frameworks advocated by Hayes (30), non-parametric bootstrap resampling (5,000 iterations) was used to estimate mediation effects and their 95% confidence intervals. All analyses were conducted on the complete dataset (*n* = 433), with the significance level set at *α* = 0.05 (two-sided).

Although the Baron-Kenny stepwise regression approach is a recognized historical method for mediation analysis, we acknowledge its relatively lower sensitivity for detecting indirect effects compared to modern resampling-based mediation frameworks established by Hayes (30) via the widely adopted PROCESS modeling paradigm. Therefore, we primarily based our inference on non-parametric bootstrap resampling (5,000 iterations) with bias-corrected accelerated 95% confidence intervals, the gold-standard inferential strategy recommended in contemporary mediation analytic guidelines for simple mediation Model 4. The significance of the indirect effect was determined by whether the confidence interval excluded zero, consistent with modern mediation reporting standards. All mediation analyses were performed using the ‘mediation’ package in R, which implements bootstrap indirect effect estimation aligned with Hayes’ regression-based conditional process analytic logic.

To control for potential confounding, we included all candidate covariates (covering demographic and clinical characteristics, such as age, education and disease severity) and performed Least Absolute Shrinkage and Selection Operator (LASSO) regression for covariate selection. LASSO was chosen over traditional stepwise or theory-driven selection because it performs data-driven regularization, reducing the risk of overfitting and model instability when handling multiple correlated covariates, especially in the absence of strong prior evidence regarding which specific confounders to retain. All covariates were standardized prior to model fitting to ensure equal penalty application. The LASSO model was estimated using the glmnet package in R, with the tuning parameter λ selected via 10-fold cross-validation using the one-standard-error rule (λ.1se). The resulting λ.1se value was 0.032. Under this penalty, all covariate coefficients were shrunk to zero, indicating that none of the candidate covariates exceeded the marginal benefit threshold for inclusion in the mediation model (The complete results with all covariates are compiled in Supplementary Table 1). This outcome suggests that the association between patient activation, self-efficacy, and self-management remained robust after accounting for these measured confounders. Consequently, all covariates were excluded from the subsequent mediation analysis to enhance model parsimony and avoid unnecessary complexity. Detailed LASSO results, including the cross-validation curve and coefficient paths, are provided in “Supplementary Document-Appendix and Supplementary Document-LASSO.” Although LASSO selected no covariates statistically, clinically relevant variables (e.g., age, education, comorbidities) were still considered in subgroup analyses to ensure clinical interpretability. This two-step approach balances statistical parsimony with clinical relevance. The raw data and the original analysis code were provided as Supplementary materials (see documents: R syntax.R and mediating_effect_1.csv), which can be directly used to reproduce the results.

Although all core variables were collected via self-report questionnaires, we implemented several procedural remedies during data collection to minimize common method bias, including ensuring anonymity, using validated scales with different response formats, and explicitly stating that there were no right or wrong answers. In addition, we conducted Harman’s single-factor test. The first unrotated factor accounted for 52% of the total variance. Although this value slightly exceeds the conventional 40% threshold, the limited number of core constructs (three) may inflate the first factor’s contribution. Considering both the procedural and statistical evidence, we concluded that common method bias did not seriously compromise the validity of our findings. Detailed results are available in Supplementary Table 2.

The primary analyses assumed linear relationships between the independent, mediator, and dependent variables. To verify this assumption, we conducted a supplementary one-way ANOVA comparing the mean scores of eHealth literacy and the combined self-efficacy/self-management measure across the four PAM-13 levels (ordinal variable). The results supported a progressive increase in outcomes across levels (*p* < 0.001), indicating that the linearity assumption was reasonable. Detailed ANOVA analysis was provided in Supplementary materials and see Supplementary-ANOVA.

## 3 Results

### 3.1 General characteristics

Of 433 participants, 62.82% were aged ≥60 years, 64.20% had a Middle school education or lower, and 79.91% were married. Most participants (56.35%) had ≤1 CHD-related healthcare visit in the past year; 78.29% had no prior cardiac stent implantation; 60.74% had a CHD duration ≤3 years; and 75.52% had at least one comorbidity. Among these characteristics, age, education, employment status, marital status, BMI, family history of CVD, number of CHD-related visits, CHD duration, cardiac functional class, and coronary stent count were considered as candidate covariates in the subsequent LASSO regression with inclusion criteria *p* < 0.05 (see Methods). Full demographic and clinical details are in Table 1.

**Table 1:** General characteristics of study subjects (*N* = 433).

General characteristics	Total (*n* = 433)	Male (*n* = 235)	Female (*n* = 198)	*P*
Age, *n* (%)				<0.001
<45 years	73(16.86)	20(8.51)	53(26.77)	
45–59 years	88(20.32)	50(21.28)	38(19.19)	
60–74 years	168(38.80)	103(43.83)	65(32.83)	
≥75 years	104(24.02)	62(26.38)	42(21.21)	
Education, *n* (%)				<0.001
≤Elementary	148(34.18)	81(34.47)	67(33.84)	
Middle school	130(30.02)	77(32.77)	53(26.77)	
High school	64(14.78)	47(20.00)	17(8.59)	
College	30(6.93)	13(5.53)	17(8.59)	
≥Bachelor’s degree	61(14.09)	17(7.23)	44(22.22)	
Employment status, *n* (%)				0.002
Employed	121(27.94)	50(21.28)	71(35.86)	
Retired	144(33.26)	89(37.87)	55(27.78)	
Other	168(38.80)	96(40.85)	72(36.36)	
Marital status, *n* (%)				0.115
Never married	27(6.24)	13(5.53)	14(7.17)	
Married	346(79.91)	196(83.40)	150(75.76)	
Divorced	17(3.93)	5(2.13)	12(6.06)	
Widowed	43(9.93)	21(8.94)	22(11.11)	
BMI, *n* (%)				0.014
Underweight (<18.50)	23(5.31)	10(4.26)	13(6.57)	
Normal (18.50–23.99)	248(57.27)	121(51.49)	127(64.14)	
Overweight (24.00–27.99)	126(29.10)	82(34.89)	44(22.22)	
Obese (≥28.00)	36(8.31)	22(9.36)	14(7.07)	
Smoking status, *n* (%)				<0.001
Current smoker	97(22.40)	86(36.60)	11(5.56)	
Former smoker	97(22.40)	89(37.87)	8(4.04)	
Never smoker	239(55.20)	60(25.53)	179(90.40)	
Living arrangement, *n* (%)				0.666
Living alone	60(13.86)	32(13.62)	28(14.14)	
Living with spouse	264(60.97)	149(63.40)	115(58.08)	
Living with children	96(22.17)	48(20.43)	48(24.24)	
Living with other caregivers	13(3.00)	6(2.55)	7(3.54)	
Household income, *n* (%)				0.609
<2000	97(22.40)	55(23.40)	42(21.21)	
2000–2,999	70(16.17)	40(17.02)	30(15.15)	
3,000–3,999	108(24.94)	56(23.83)	52(26.26)	
4,000–4,999	58(13.39)	35(14.89)	23(11.62)	
≥5,000	100(23.09)	49(20.85)	51(25.76)	
Medical insurance, *n* (%)				0.209
Urban and Rural Resident Basic Medical Insurance	247(57.04)	141(60.00)	106(53.54)	
Urban Employee Basic Medical Insurance	186(42.96)	94(40.00)	92(46.46)	
Past-year CHD healthcare visits, *n* (%)				0.043
≤1	244(56.35)	120(51.06)	124(62.63)	
2–3	143(33.03)	85(36.17)	58(29.29)	
>3	46(10.62)	30(12.77)	16(8.08)	
Cardiac functional class, *n* (%)				<0.001
Class I	164(37.88)	76(32.34)	88(44.44)	
Class II	158(36.49)	88(37.45)	70(35.35)	
Class III	87(20.09)	49(20.85)	38(19.19)	
Class IV	24(5.54)	22(9.36)	2(1.01)	
Coronary stent count, *n* (%)				<0.001
None	339(78.29)	166(70.64)	173(87.37)	
1	46(10.62)	35(14.89)	11(5.56)	
2	33(7.62)	24(10.21)	9(4.55)	
≥3	15(3.46)	10(4.26)	5(2.53)	
CHD duration, *n* (%)				0.034
≤3 years	263(60.74)	129(54.89)	134(67.68)	
4–6 years	88(20.32)	53(22.55)	35(17.68)	
7–10 years	62(14.32)	39(16.60)	23(11.62)	
11–19 years	15(3.46)	9(3.83)	6(3.03)	
≥20 years	5(1.15)	5(2.13)	0(0.0)	
Comorbidities, *n* (%)				0.007
Yes	327(75.52)	190(80.85)	137(69.19)	
No	106(24.48)	45(19.15)	61(30.81)	
Family history of CVD, *n* (%)				0.665
Yes	143(33.03)	75(31.91)	68(34.34)	
No	290(66.97)	160(68.09)	130(65.66)	

### 3.2 Scores for patient activation, eHealth literacy, self-efficacy and self-management

The mean patient activation score was 58.89 (SD = 18.87). The mean eHealth literacy score was 24.92 (SD = 8.94). The combined self-efficacy and self-management measure averaged 60.70 (SD = 15.34). Interpretation of these means was based on established scale ranges and cutoffs. For the PAM-13, scores are categorized as Level 1 (≤47.0), Level 2 (47.1–55.1), Level 3 (55.2–67.0), and Level 4 (≥67.1); the observed mean of 58.89 corresponds to Level 3, indicating moderate activation. The eHEALS total score ranges from 8 to 40, with higher scores reflecting greater literacy; the mean of 24.92 lies near the scale midpoint, suggesting a moderate level. For the HH-SESM (total range 24–96), scores ≥36, 25–35, and ≤24 represent high, moderate, and low levels, respectively; the mean of 60.70 falls well above the moderate threshold, indicating moderate-to-high self-efficacy and self-management. Subscale scores appear in Table 2.

**Table 2:** Scores of study variables (*N* = 433).

Scale	Number of scale	Score range	Score, mean (SD)
Patient activation	13	0–100	58.89 ± 18.87
eHealth literacy	8	8–40	24.92 ± 8.94
Self-efficacy and self-management	12	24–96	60.70 ± 15.34
Self-efficacy	12	12–48	30.26 ± 7.92
Self-management	12	12–48	30.43 ± 7.75

### 3.3 Disparities in eHealth literacy scores across key demographic subgroups

eHealth literacy scores differed significantly across subgroups. Higher scores were seen in younger patients, those with higher education, and those with high Monthly Income (all *p* < 0.01). The steepest gradient was by education: patients with a college degree or above scored markedly higher than those with primary education or less. A similar but less pronounced inverse gradient was observed with increasing age. To quantify the magnitude of these disparities, mean differences and standardized effect sizes (Cohen’s d) for highest vs. lowest value comparisons are reported in Table 3. For instance, the education-based contrast showed a large effect (*d* > 0.8), whereas age-related differences were of medium size (*d* = 0.66) (Table 3).

**Table 3:** eHealth literacy scores across demographic subgroups (*N* = 433).

Characteristic	Subgroup	n(%)/Category	eHealth literacy score, Mean ± SD	*p*-value*	Extreme group contrast (highest vs. lowest) Mean difference, Cohen’s *d*
Age group	<45 years	73(16.9%)	28.4 ± 9.2	<0.001	≥75 vs. < 45: −5.6, *d* = 0.66
	45–59 years	88(20.3%)	26.1 ± 8.5		
	60–74 years	168(38.8%)	24.3 ± 8.1		
	≥75 years	104(24.0%)	22.8 ± 7.9		
Education level	Primary or below	148(34.2%)	21.5 ± 7.8	<0.001	College or above vs. Primary: 8.7, *d* = 1.05
	Middle School	130(30.0%)	23.9 ± 8.2		
	High School	64(14.8%)	26.8 ± 8.6		
	College or above	91(21.0%)	30.2 ± 9.1		
Employment status	Employed	121(28.0%)	27.6 ± 9.0	0.002	Employed vs. Other: 4.1, *d* = 0.48
	Retired	144(33.3%)	24.8 ± 8.4		
	Other (e.g., Unemployed)	168(38.8%)	23.5 ± 8.1		
Household monthly income	<2000	97(22.4%)	21.9 ± 7.5	<0.001	≥5,000 vs. < 2000: 7.2, *d* = 0.85
	2000–3,999	178(41.1%)	24.3 ± 8.2		
	4,000–4,999	58(13.4%)	26.7 ± 8.9		
	≥5,000	100(23.1%)	29.1 ± 9.3		
Smoking status	Current Smoker	97(22.4%)	24.1 ± 8.3	0.045	Never vs. Current: 2.7, *d* = 0.31
	Former Smoker	97(22.4%)	25.3 ± 8.6		
	Never Smoker	239(55.2%)	26.8 ± 9.0		

*p*-values derived from ANOVA or independent *t*-test as appropriate.

### 3.4 Correlation analysis of patient activation, eHealth literacy, self-efficacy and self-management

Pearson correlation analysis (Table 4) showed moderate positive correlations between patient activation and eHealth literacy (*r* = 0.51, *p* < 0.001) and between patient activation, self-efficacy and self-management (*r* = 0.59, *p* < 0.001), and a weak positive correlation between eHealth literacy and self-efficacy and self-management (*r* = 0.37, *p* < 0.001).

**Table 4:** Correlation results.

Scale	Patient activation	eHealth literacy	Self-efficacy and self-management
Patient activation	1.000		
eHealth literacy	0.51***	1.000	
Self-efficacy and self-management	0.59***	0.37***	1.000

***p < 0.001 (two-tailed, FDR-corrected).

### 3.5 The mediating role of eHealth literacy between patient activation and self-efficacy and self-management

Patient activation (X) was specified as the independent variable, self-efficacy and self-management (Y) as the dependent variable and eHealth literacy (M) as the mediator. To maintain clarity and consistency, unstandardized regression coefficients (B) are primarily reported, with standardized coefficients (β) shown in parentheses for comparability.

Patient activation had a total positive effect on self-efficacy and self-management (*β* = 0.585, *p* < 0.001), as well as on eHealth literacy (*β* = 0.508, *p* < 0.001). When patient activation and eHealth literacy jointly predicted self-efficacy and self-management, patient activation remained positively associated with self-efficacy and self-management (*β* = 0.533, *p* < 0.001), and eHealth literacy was also positively associated with it (*β* = 0.103, *p* = 0.023).

**Figure 1:**
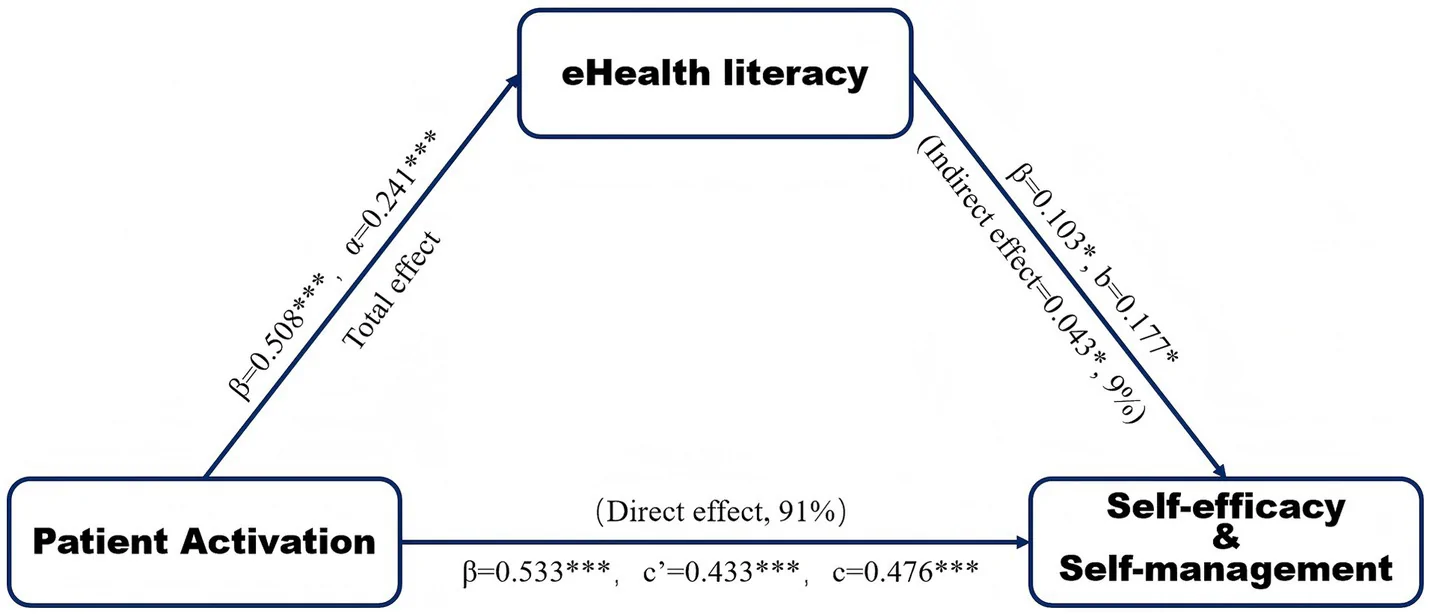
Mediation model. This figure depicts the partial mediating role of eHealth literacy in the relationship between patient activation and self-efficacy and self-management among coronary heart disease patients. Patient activation exerts a direct positive effect on self-efficacy and self-management (*β* = 0.533, *p* < 0.001), accounting for 91% of the total effect. Meanwhile, patient activation positively predicts eHealth literacy (*β* = 0.508, *p* < 0.001), and eHealth literacy further positively influences self-efficacy and self-management (*β* = 0.103, *p* < 0.05), forming an indirect effect (*β* = 0.043, *p* < 0.05) that accounts for 9% of the total effect. The total effect of patient activation on self-efficacy and self-management is significant (*β* = 0.585, *p* < 0.001), confirming eHealth literacy as a partial mediator in this relationship.

Bootstrap analysis (5,000 resamples) with bias-corrected accelerated 95% confidence intervals yielded an indirect effect (a × b) of *B* = 0.043, 95% CI [0.002, 0.088] (*β* = 0.052). Because the confidence interval excluded zero, a significant partial mediation was established. The proportion of the total effect mediated by eHealth literacy was calculated as (indirect effect / total effect) × 100% using the unstandardized coefficients: 0.043/0.476 = 9.0%. Accordingly, the direct effect accounted for the remaining 91% of the total effect. Although the indirect pathway was statistically significant, the mediated proportion was small, indicating that eHealth literacy plays a minor mediating role and that most of the association between patient activation and self-efficacy and self-management operates through other mechanisms or remains direct (Tables 5, 6 and Figure 1).

**Table 5:** Regression analysis of the mediation model.

Path model	Independent variable	Dependent variable	*R* ^2^	B	*β*	SE	*P*
X-Y	Patient activation	Self-efficacy and self-management	0.343	0.476(*c*)	0.585	0.032	<0.001***
X-M	Patient activation	eHealth literacy	0.258	0.241(*a*)	0.508	0.020	<0.001***
X、M-Y	Patient activation	Self-efficacy and self-management	0.350	0.433(*c’*)	0.533	0.037	<0.001***
	eHealth literacy			0.177(*b*)	0.103	0.077	0.023*

R^2^ = Coefficient of determination, B = Unstandardized coefficient, β = Standardized coefficient, SE = Standard error. ***p < 0.001.

**Table 6:** Mediation effect test.

Effect type	*B*	95%CI	*P*	Proportion of total effect (%)
Total effect	0.476(*c*)	[0.411, 0.538]	<0.001***	—
Direct effect	0.433(*c’*)	[0.356, 0.508]	<0.001***	91%
Indirect effect	0.043(*a × b*)	[0.002, 0.088]	0.043*	9%

B = Unstandardized coefficient, 95% CI = 95% Confidence Interval. ***p < 0.001, *p < 0.05.

### 3.6 Subgroup analysis for the mediation effect by age and education

To verify result robustness, we performed subgroup analyses based on age and educational level, two common influential covariates. No significant interaction effects of age (*p* = 0.188) and education (*p* = 0.120) were detected for the mediating effect. A statistically significant mediating association was only observed in participants younger than 60 years, whereas no evident mediating effect existed in the group of older adults. No significant mediating difference was found across educational subgroups (Supplementary Table 3).

## 4 Discussion

### 4.1 Current status of patient activation, eHealth literacy, self-efficacy and self-management

The mean patient activation score of 58.89 corresponds to PAM-13 Level 3, indicating that individuals are taking action but may lack confidence and skills for sustained self-management (31). This modest activation level likely reflects the older age and lower education profile of the sample, both of which are known correlates of lower activation (32, 33). For CHD patients in similar settings, limited health literacy and disease-related fatigue may further impede activation.

eHealth literacy was moderately low (mean 24.92), consistent with studies in older and less-educated populations (34), and illustrates the second-level digital divide in this group. Self-efficacy and self-management scores were moderate, and may be linked to the ongoing burden of chronic illness that diminishes positive affect and motivation (35–37). These findings underscore the need to address both psychological and skill-based determinants to support self-management in CHD patients.

### 4.2 Key demographic determinants of eHealth literacy

Findings align with the digital divide framework, where disparities in access, skills, and beneficial use of technology intersect (38–40). The strong educational gradient reflects that foundational literacy and cognitive resources, core to the second-level divide, are essential for critically appraising digital health content (41). The age gradient likely stems from lower digital familiarity and confidence, as well as age-related cognitive changes (34, 35). Lower income further compounds disadvantage, limiting device and broadband access (first-level divide) and reducing opportunities to develop eHealth skills, thereby reinforcing health inequities (42, 43).

These patterns also help explain the lower patient activation observed in the sample: older, less-educated, and lower-income patients face concurrent barriers to both engagement in care and digital health use, potentially creating a mutually reinforcing cycle of disadvantage. This intersection highlights the need to embed eHealth literacy support within activation-enhancing interventions.

### 4.3 Correlation analysis of patient activation, eHealth literacy, self-efficacy and self-management in coronary heart disease patients

The moderate patient activation–eHealth literacy correlation (*r* = 0.51) is comparable to previous reports in chronic disease populations (44), suggesting that more activated patients tend to seek and engage with digital health information, which in turn may reinforce activation (45, 46). The moderate activation–self-management correlation (*r* = 0.59) is consistent with evidence linking activation with better self-care behaviors (47–49). The weaker eHealth literacy–self-management association (*r* = 0.37) mirrors prior work (50, 51), indicating that while eHealth literacy can facilitate access to support networks and health knowledge, its direct contribution to self-management behaviors is modest and likely depends on additional factors such as motivation and social support (52).

### 4.4 Theoretical interpretation and the mediating role of eHealth literacy

Drawing on Social Cognitive Theory, patient activation reflects self-regulatory agency, while eHealth literacy can be viewed as an environmental enabler within the digital health context, both contributing to self-efficacy and self-management behaviors. The partial mediation model is consistent with this framework: patient activation not only directly fosters confidence and self-care but also operates indirectly by promoting the capability to use digital resources for health.

The direct pathway was substantial, whereas the indirect effect via eHealth literacy, although statistically significant, accounted for only 9% of the total effect. This modest mediation suggests that improving eHealth literacy alone may yield limited additional benefit in self-management outcomes unless combined with strategies targeting activation and other psychosocial determinants. In the context of an older, less-educated CHD population, this finding holds theoretical importance in clarifying a mechanism, but its clinical significance should be interpreted cautiously. The small effect size likely reflects that digital engagement is only one of many resources for self-management, and that unmeasured factors—such as social support, depression, or health anxiety—may play larger roles (23-item comment).

While the 9% mediated proportion was statistically significant (as evidenced by the bias-corrected 95% CI excluding zero), this statistical significance should not be equated with clinical or practical importance. From a clinical standpoint, the small effect size implies that even a marked improvement in eHealth literacy would translate into only marginal gains in patients’ self-efficacy and self-management—given that over 90% of the total association was driven directly by patient activation itself. Consequently, in resource-constrained cardiac rehabilitation or nursing practice, interventions primarily targeting patient activation (e.g., motivational interviewing, collaborative goal-setting, and confidence-building exercises) are likely to yield substantially larger and more tangible benefits for self-management than interventions focused solely on enhancing eHealth literacy. The latter should be viewed as a complementary rather than a primary therapeutic strategy, best integrated into broader activation-enhancing programs rather than deployed as a standalone solution.

### 4.5 Comparison with previous studies and novelty

In CHD populations, studies examining activation, eHealth literacy, and self-management together are scarce. Most prior work on eHealth literacy has focused on community-dwelling adults or mixed chronic disease samples. The present study extends this literature by testing a mediation model specifically in hospitalized CHD patients from a developing country context, where digital health infrastructure is expanding but unevenly accessible. Unlike studies that treat eHealth literacy solely as a predictor, we positioned it as a mediator, integrating digital literacy within cardiovascular self-management frameworks. This approach provides initial evidence that the patient activation–self-management relationship partly operates through digital health capability, though cross-sectional data limit causal inference.

Beyond traditional eHealth literacy, the rise of AI-driven cardiac tools demands AI literacy—the capacity to critically evaluate automated recommendations and algorithmic biases (53, 54). Our sample’s marked educational and age gradients in eHealth literacy suggest these same subgroups face even steeper barriers to AI-facilitated self-management. Consistent with the digital inclusion and telehealth inequity literature, smartphone access alone does not guarantee benefit; digital readiness, confidence, and support are stronger predictors of engagement (55, 56). The modest mediating role of eHealth literacy (9%) implies that isolated AI-literacy training may yield limited gains unless combined with patient activation and psychosocial support. Clinically, cardiac rehabilitation should screen for digital readiness and offer tiered support—from simplified interfaces to offline alternatives—while health systems must invest in equity-oriented telehealth policies. Future longitudinal research should evaluate whether AI-literacy interventions effectively reduce disparities in CHD self-management (53–56).

### 4.6 Limitations and future directions

Several limitations merit consideration. First, the convenience sample drawn from a single tertiary hospital in Hunan Province, with an overrepresentation of older and less-educated patients, introduces potential selection bias and limits external validity; findings may not generalize to community settings or younger CHD cohorts. Future studies should adopt multicenter recruitment or probability sampling to enhance generalizability. Second, additionally, this study did not collect detailed clinical information, including CHD severity (e.g., number of diseased vessels, left ventricular function), specific comorbidities beyond a simple count, history of cardiac interventions (e.g., percutaneous coronary intervention, coronary artery bypass grafting), or major pharmacological therapies (e.g., dual antiplatelet therapy, anticoagulants). The absence of these clinical variables limits our ability to adjust for disease-related confounding or to examine whether the mediation model differs across clinical subgroups. Third, we did not assess potential confounders such as depressive symptoms, social support, or health anxiety, which may influence self-management; their inclusion in future multivariable models is necessary to disentangle independent predictors of eHealth literacy. Forth, since our study was designed around patient self-reported questionnaire data, the objective clinical metrics (e.g., hypertension, diabetes, dyslipidemia, prior PCI/CABG, and major cardiovascular medications) were not collected, preventing their inclusion in our analysis. This limitation may restrict our ability to fully evaluate the impact of these factors or conduct subgroup analyses. Future research will incorporate these objective clinical indicators to enhance the clinical relevance and applicability of our findings. Fifth, the cross-sectional design captures associations at a single time point and cannot evaluate longitudinal changes in self-management or patient activation. Critically, although bootstrapping was used for mediation, the findings reflect statistical associations and cannot be interpreted causally. Cross-sectional mediation analysis cannot establish temporal precedence and may overestimate indirect effects; thus, the mediation results should be viewed with caution. To strengthen temporal and causal inference, future research should employ longitudinal or prospective cohort designs. Such designs, combined with structural equation modeling or path analysis, would permit mediation testing with proper temporal ordering, correction for measurement error, and examination of more complex pathways. Ultimately, intervention studies are required to verify whether enhancing eHealth literacy and patient activation translates into clinically meaningful improvements in self-management. Emerging approaches such as AI-assisted patient education, mobile cardiac rehabilitation, and telemonitoring could be integrated into these interventions, but their effectiveness for patients with low baseline eHealth literacy remains to be tested. Although all instruments used in this study (PAM-13, eHEALS, and HH-SESM) have demonstrated acceptable reliability in prior studies and in our sample (Cronbach‘s α ranging from 0.81 to 0.88), we did not perform confirmatory factor analysis (CFA) to specifically evaluate their construct validity within this CHD population. Future research should therefore conduct CFA or multi-group CFA to assess measurement invariance and construct validity of these scales across different CHD subgroups (e.g., by age, education, or disease severity), thereby strengthening the psychometric evidence base for their use in this population.

### 4.7 Clinical and policy implications

Despite limitations, the findings offer actionable insights. First, cardiac rehabilitation and nursing discharge planning should incorporate brief eHealth literacy screening, especially for older adults (≥65 years) with less than high school education and low household income, as this subgroup exhibited the lowest activation and eHealth literacy in our sample. For those with low eHealth literacy, a tiered intervention strategy is recommended based on digital readiness: (i) patients with no smartphone experience could receive automated voice calls (interactive voice response) or SMS-based health tips using simple pictograms to support medication adherence and symptom monitoring; (ii) those with basic smartphone skills could be introduced to a simplified cardiac rehabilitation application with large-font, audio-narrated instructions and minimal navigation steps. Delivery should be through face-to-face training by community health workers or nurses during home visits or routine follow-up appointments, followed by weekly booster phone calls to reinforce skills and troubleshoot problems. Offline alternatives, such as printed self-management booklets and telephone coaching, must be maintained to avoid excluding those unable or unwilling to engage digitally. Second, interventions that simultaneously build patient activation and eHealth literacy may yield greater gains in self-management than single-component approaches. Our mediation findings indicate that patients with moderate activation but low eHealth literacy, as well as those with coexisting low activation and low eHealth literacy, are the groups most likely to benefit from integrated programs. An example of such a program is a nurse-led, small-group intervention combining brief motivational interviewing to enhance activation with hands-on practice in using a blood pressure telemonitoring app and accessing a patient portal. For rural or mobility-limited patients, this intervention could be adapted for delivery via videoconferencing. Third, health systems in developing regions should invest in user-centered design and broadband equity, for instance by subsidizing mobile data plans for low-income cardiac patients and funding community-based digital navigators to assist with device setup and initial training. These measures are essential to ensure that digital health transformation does not exacerbate existing disparities in CHD outcomes. These recommendations remain tentative pending longitudinal evidence.

## 5 Conclusion

Patient activation, eHealth literacy, self-efficacy and self-management in patients with CHD were found to be at moderate levels. Substantial disparities in eHealth literacy were identified across age, education, employment and income groups, indicating that older, less educated and socioeconomically disadvantaged patients may face greater challenges in engaging with digital health resources and could be considered priority populations for supportive strategies; however, the cross-sectional design precludes causal conclusions about the impact of such support. Correlation analyses revealed generally moderate positive associations among the variables, with eHealth literacy showing a weaker positive correlation with self-efficacy and self-management. eHealth literacy partially mediated the relationship between patient activation and both self-efficacy and self-management as separate constructs, accounting for approximately 9% of the total effect. Although statistically significant, this modest effect size suggests that the indirect pathway through eHealth literacy, while theoretically relevant, may have limited practical impact in isolation, and caution is warranted in interpreting these findings as evidence for intervention targets. The observed cross-sectional pattern is consistent with the hypothesis that higher patient activation may be associated with greater use of online health information, which in turn may be linked to stronger self-efficacy and self-management behaviors; nevertheless, these associations do not establish that deliberately improving patient activation or eHealth literacy will cause gains in self-efficacy or self-management. By demonstrating the mediating role of eHealth literacy, this study extends patient activation and digital health literacy models within CHD management and offers a specific theoretical contribution to digital health-based cardiovascular nursing. The findings suggest that strategies such as simplified eHealth interfaces, nurse-assisted digital coaching and low-tech alternatives could be explored in future intervention development to address digital disparities among vulnerable CHD patients; however, their effectiveness has not been evaluated, and they should not be considered evidence-based recommendations for immediate practice. Generalizability is limited by the single-center convenience sample and the specific demographic profile, and interpretations for other populations should be made cautiously. Longitudinal studies with repeated measures are needed to establish temporal relationships and test the hypothesized causal pathways, while randomized intervention trials are required to determine whether programs targeting both patient activation and eHealth literacy can effectively improve self-efficacy and self-management in this population. Finally, while the Baron-Kenny stepwise regression approach has lower sensitivity for detecting indirect effects, we supplemented it with non-parametric bootstrap resampling to obtain robust confidence intervals.

## Data Availability

The original contributions presented in the study are included in the article/Supplementary material, further inquiries can be directed to the corresponding author/s.

## References

[1] Chu C Liu S Nie L Hu H Liu Y Yang J. The interactions and biological pathways among metabolomics products of patients with coronary heart disease. Biomed Pharmacother. (2024) 173:116305. doi: 10.1016/j.biopha.2024.116305 38422653

[2] Guo J Wang H Li Y Zhu S Hu H Gu Z. Nanotechnology in coronary heart disease. Acta Biomater. (2023) 171:37–67. doi: 10.1016/j.actbio.2023.09.011, 37714246

[3] Mi Y Xue Z Qu S Yin Y Huang J Kou R . The economic burden of coronary heart disease in mainland China. Public Health (Lond). (2023) 224:140–51. doi: 10.1016/j.puhe.2023.08.034, 37797560

[4] Diseases N C F C, China T W C O. Report on cardiovascular health and diseases in China 2024:an updated summary. Chin Circ J. (2025) 40:521–59. doi: 10.1211/ccj.184

[5] Lu M Hravnak M Chang Y Lin Y Zhang X Ma J . Patterns of adherence to secondary prevention measures among Chinese patients with coronary artery disease: a longitudinal study. J Cardiovasc Nurs. (2022) 37:E61–72. doi: 10.1097/JCN.0000000000000830, 34238840

[6] Su JJ Yu DS. Effects of a nurse-led eHealth cardiac rehabilitation programme on health outcomes of patients with coronary heart disease: a randomised controlled trial. Int J Nurs Stud. (2021) 122:104040. doi: 10.1016/j.ijnurstu.2021.104040, 34333211

[7] Sigamani A Gupta R. Revisiting secondary prevention in coronary heart disease. Indian Heart J. (2022) 74:431–40. doi: 10.1016/j.ihj.2022.11.011, 36455667 PMC9773289

[8] Sugiharto F Nuraeni A Trisyani Y Putri A Armansyah N Zamroni A. A scoping review of predictors associated with self-efficacy among patients with coronary heart disease. Vasc Health Risk Manag. (2023) 19:719–31. doi: 10.2147/VHRM.S435288, 37965056 PMC10642341

[9] Hibbard JH Mahoney ER Stockard J Tusler M. Development and testing of a short form of the patient activation measure. Health Serv Res. (2005) 40:1918–30. doi: 10.1111/j.1475-6773.2005.00438.x, 16336556 PMC1361231

[10] Hernar I Graue M Igland J Richards DA Riise HKR Haugstvedt A . Patient activation in adults attending appointments in general practice: a cross-sectional study. BMC Prim Care. (2023) 24:144. doi: 10.1186/s12875-023-02102-9, 37430197 PMC10331983

[11] Son YJ Lee SJ Kim SH. The role of patient activation in self-care behaviours of Korean older adults with heart failure. Nurs Open. (2023) 10:5202–10. doi: 10.1002/nop2.1756, 37013816 PMC10333907

[12] Hussein WF Bennett PN Abra G Watson E Schiller B. Integrating patient activation into dialysis care. Am J Kidney Dis. (2022) 79:105–12. doi: 10.1053/j.ajkd.2021.07.015, 34461165

[13] Lunardi LE Hill K Xu Q Le Leu R Bennett PN. The effectiveness of patient activation interventions in adults with chronic kidney disease: a systematic review and meta-analysis. Worldviews Evid-Based Nurs. (2023) 20:238–58. doi: 10.1111/wvn.12634, 36906914

[14] Wang Y Masingboon K Wacharasin C. Mediating role of self-efficacy in the relationship between family functioning and self-management behaviors in patients with coronary heart disease: a cross-sectional study in Jiangsu, China. Belitung Nurs J. (2025) 11:59–66. doi: 10.33546/bnj.3638, 39877215 PMC11770265

[15] Zhang X Chen H Liu Y Yang B. Influence of chronic illness resources on self-management and the mediating effect of patient activation among patients with coronary heart disease. Nurs Open. (2021) 8:3181–9. doi: 10.1002/nop2.1031 PMC8510723 34498405

[16] Zhu Y Song Y Wang Y Ji H Wang D Cai S . Relationships among social support, self-efficacy, and patient activation in community-dwelling older adults living with coronary heart disease: a cross-sectional study. Geriatr Nurs. (2022) 48:139–44. doi: 10.1016/j.gerinurse.2022.09.008 36219932

[17] Milanti A Norman C Chan DNS So WKW Skinner H. eHealth literacy 3.0: updating the Norman and Skinner 2006 model. J Med Internet Res. (2025) 27:e70112. doi: 10.2196/70112, 40068166 PMC11937704

[18] El Benny M Kabakian-Khasholian T El-Jardali F Bardus M. Application of the eHealth literacy model in digital health interventions: scoping review. J Med Internet Res. (2021) 23:e23473. doi: 10.2196/23473, 34081023 PMC8212628

[19] Hua Z Yuqing S Qianwen L Hong C. Factors influencing eHealth literacy worldwide: systematic review and Meta-analysis. J Med Internet Res. (2025) 27:e50313. doi: 10.2196/50313, 40063939 PMC11933766

[20] Délétroz C Allen MC Sasseville M Rouquette A Bodenmann P Gagnon MP. eHealth literacy measurement tools: a systematic review protocol. Syst Rev. (2022) 11:205. doi: 10.1186/s13643-022-02076-2, 36151577 PMC9508732

[21] Writing Committee Members Lawton JS Tamis-Holland JE Bangalore S Bates ER Beckie TM . 2021 ACC/AHA/SCAI Guideline for Coronary Artery Revascularization: A Report of the American College of Cardiology/American Heart Association Joint Committee on Clinical Practice Guidelines. J Am Coll Cardiol. (2021) 79:e21– e129. doi: 10.1016/j.jacc.2021.09.006, 34895950

[22] Ping N Jingli C Na L. The sample size estimation in quantitative nursing research. Chin J Nurs. (2010) 45:378–80. doi: 10.1307/CJN.0000000000001930

[23] Brenk-Franz K Hibbard JH Herrmann WJ Freund T Szecsenyi J Djalali S . Validation of the German version of the patient activation measure 13 (PAM13-D) in an international multicentre study of primary care patients. PLoS One. (2013) 8:e74786. doi: 10.1371/journal.pone.0074786, 24098669 PMC3787015

[24] Ng QX Liau MYQ Tan YY Tang ASP Ong C Thumboo J . A systematic review of the reliability and validity of the patient activation measure tool. Healthcare (Basel). (2024) 12:1079. doi: 10.3390/healthcare12111079, 38891154 PMC11171848

[25] Tsujimoto T Abe T Kuroda Y Yamasaki M Isomura M. Reliability and validity of the Japanese version of the eHealth literacy scale in community-dwelling older adults: a cross-sectional study. Eur J Investig Health Psychol Educ. (2025) 16:1. doi: 10.3390/ejihpe16010001, 41590011 PMC12839868

[26] Mares M Salamonson Y Maneze D Elmir R Everett B. Development and validation of a scale to measure self-efficacy and self-management in people with coronary heart disease. J Cardiovasc Nurs. (2022) 37:E81–8. doi: 10.1097/JCN.0000000000000777, 37707975

[27] Mi S Yang R Wang H. Reliability and validity of the Chinese version of the heart health self-efficacy and self-management scale in patients with coronary heart disease. Chin J Nurs. (2021) 56:378–83. doi: 10.1307/CJN.0000000000001356

[28] Tunc Suygun E Vardar Yagli N Suygun H Saglam M. Validity and reliability of the Turkish translation of the heart health self-efficacy and self-management scale in patients with chronic heart disease. BMC Psychol. (2025) 13:1210. doi: 10.1186/s40359-025-03539-6, 41185050 PMC12581388

[29] Baron RM Kenny DA. The moderator-mediator variable distinction in social psychological research: conceptual, strategic, and statistical considerations. J Pers Soc Psychol. (1986) 51:1173–82. doi: 10.1037//0022-3514.51.6.1173, 3806354

[30] Hayes AF. Introduction to Mediation, Moderation, and Conditional Process Analysis: A Regression-Based Approach. 3rd ed. New York: The Guilford Press (2022).

[31] Dammery G Vitangcol K Ansell J Ellis LA Smith CL Carrigan A . The patient activation measure (PAM) and the pandemic: predictors of patient activation among Australian health consumers during the COVID-19 pandemic. Health Expect. (2023) 26:1107–17. doi: 10.1111/hex.13725, 36810854 PMC10154866

[32] Chan EY Glass GF Jr Cheong RQ Chin GF Chng DYJ. Patient activation and its predictors in hospitalized older adults in Singapore. Geriatr Nurs. (2021) 42:336–43. doi: 10.1016/j.gerinurse.2021.01.006, 33556900

[33] Innab A Kerari A Alqahtani N Albloushi M Alshammari A. Patient activation, adherence to hypertension treatment plans and blood pressure control in Saudi Arabia: a cross-sectional study. BMJ Open. (2023) 13:e067862. doi: 10.1136/bmjopen-2022-067862, 36697049 PMC9884875

[34] Bäuerle A Marsall M Jahre LM Rammos C Mallien C Skoda EM . Psychometric properties of the German revised version of the eHealth literacy scale in individuals with cardiac diseases: validation and test of measurement invariance. Digit Health. (2023) 9:20552076231194915. doi: 10.1177/20552076231194915, 37588160 PMC10426311

[35] Liu S Zhao H Fu J Kong D Zhong Z Hong Y . Current status and influencing factors of digital health literacy among community-dwelling older adults in Southwest China: a cross-sectional study. BMC Public Health. (2022) 22:996. doi: 10.1186/s12889-022-13378-4, 35581565 PMC9112275

[36] Zhang S Wang Z Lin X Li Y Xue Y Ban J . Kinesiophobia and self-management behaviour related to physical activity in Chinese patients with coronary heart disease: the mediating role of self-efficacy. Nurs Open. (2023) 10:105–14. doi: 10.1002/nop2.1283, 35773943 PMC9748108

[37] Sun C Chen C Feng J Zhang T Zhang Y Pan X . Current status and factors influencing mental health literacy in patients with coronary heart disease: a cross-sectional study. Nurs Health Sci. (2024) 26:e13165. doi: 10.1111/nhs.13165, 39349355

[38] Pan TH Aung MN Moolphate S Aung TNN Koyanagi Y Hok Ka CM . Thai older persons' digital capacity and application of Technology for Healthy Aging: a mixed-method study. J Aging Res. (2025) 2025:8080561. doi: 10.1155/jare/8080561, 41356606 PMC12680461

[39] Hunsaker A Hargittai E Micheli M. Relationship between internet use and change in health status: panel study of young adults. J Med Internet Res. (2021) 23:e22051. doi: 10.2196/22051, 33439134 PMC7840280

[40] Rapson T Ramirez M He S Wong J Kim HC Luna I . Identifying skill and usability barriers to digital health tool use among older adult patients in US safety net clinics: mixed methods study. JMIR Hum Factors. (2026) 13:e78430. doi: 10.2196/78430, 42081796 PMC13138792

[41] Van Deursen AJ van Dijk JA. Internet skills and the digital divide. New Media Soc. (2021) 13:893–911. doi: 10.1036/NWS-2021-067786

[42] Fang ML Canham SL Battersby L Sixsmith J Wada M Sixsmith A. Exploring privilege in the digital divide: implications for theory, policy, and practice. The Gerontologist. (2019) 59:e1–e15. doi: 10.1093/geront/gny037, 29750241

[43] Gong E Wang H Zhu W Galea G Xu J Yan LL . Bridging the digital divide to promote prevention and control of non-communicable diseases for all in China and beyond. BMJ. (2024) 387:e076768. doi: 10.1136/bmj-2023-076768, 39424328 PMC11487297

[44] Erişen MA. Effect of health literacy and patient activation on health-seeking behaviour: a cross-sectional study in Turkey. Health Expect. (2024) 27:e70052. doi: 10.1111/hex.70052, PMC11456960 39373139

[45] Gagnon MP Ndiaye MA Larouche A Chabot G Chabot C Buyl R . Optimising patient active role with a user-centred eHealth platform (CONCERTO+) in chronic diseases management: a study protocol for a pilot cluster randomised controlled trial. BMJ Open. (2019) 9:e028554. doi: 10.1136/bmjopen-2018-028554, 30944143 PMC6500232

[46] Qiu SS Ye JF You F Liu M Zhao X. How does mHealth benefit older Chinese adults' quality of life? Examining the roles of eHealth literacy, health motivation, and patient activation. Digit Health. (2025) 11:20552076241313160. doi: 10.1177/20552076241313160, 39807427 PMC11726531

[47] Zhang A Wang J Wan X Guo Z Zhang Z Zhao S . The mediating effect of self-efficacy on the relationship between diabetes self-management ability and patient activation in older adults with type 2 diabetes. Geriatr Nurs. (2023) 51:136–42. doi: 10.1016/j.gerinurse.2023.02.017, 36940508

[48] Chen J Tian Y Yin M Lin W Tuersun Y Li L . Relationship between self-efficacy and adherence to self-management and medication among patients with chronic diseases in China: a multicentre cross-sectional study. J Psychosom Res. (2023) 164:111105. doi: 10.1016/j.jpsychores.2022.111105, 36495756

[49] Lee EH Lee YW Kang EH Kang HJ. Relationship between electronic health literacy and self-management in people with type 2 diabetes using a structural equation modeling approach. J Nurs Res. (2024) 32:e315. doi: 10.1097/jnr.0000000000000588, 38128065

[50] Qian J Yao X Liu T. Assessment of electronic health literacy and its association with self-management among gout patients: a cross-sectional study. Arch Rheumatol. (2024) 39:358–67. doi: 10.46497/ArchRheumatol.2024.10397, 39507850 PMC11537690

[51] Shiu LS Liu CY Lin CJ Shiu L‐S Liu C‐Y Lin C‐J . What are the roles of eHealth literacy and empowerment in self-management in an eHealth care context? A cross-sectional study. J Clin Nurs. (2023) 32:8043–53. doi: 10.1111/jocn.16876, 37668267

[52] Provenzano M Cillara N Podda M Gonzalez CIA Cicalò E Fransvea P . Validity and reliability of patient activation measure (PAM13-I) Italian version among patient undergoing elective surgery. Res Nurs Health. (2025) 48:281–93. doi: 10.1002/nur.22447, 39874068

[53] Biagini G. Towards an AI-literate future: a systematic literature review exploring education, ethics, and applications. Int J Artif Intell Educ. (2025) 35:2616–66. doi: 10.1007/s40593-025-00466-w

[54] Memarian B Doleck T. From digital literacy to AI literacy: a systematic review and integrative framework for education. Int J AI Pedagog Innov Learn Fut. (2026) 2. doi: 10.46787/ijaipil.v2i1.7287

[55] Boerkamp L. G. P. van Deursen A. J. A. M. van Laar E. van Deursen A.J.A.M. Zeeuw Alex Graaf Shenja . Cocreating support: toward a digital inclusion intervention for parents in poverty CoDesign (2026) 26 1–16 doi: 10.1080/15710882.2025.2608872

[56] Labadorf J Nichols M Williams T Cunanan C D'Anza B. A smartphone is not enough: telehealth attendance and the digital divide. Health Care Sci. (2025) 4:259–68. doi: 10.1002/hcs2.70033, 40861513 PMC12371716

