# Supplementary material for: Patient activation and self-management in patients with coronary heart disease: the mediating role of eHealth literacy
Source: Front Public Health. 2026 Sep 11;14:1799712. doi: 10.3389/fpubh.2026.1799712 (PMC13612537; doi:10.3389/fpubh.2026.1799712)
Supplement: Supplementary file 1 [file Data_Sheet_1.pdf]

Supplementary Materials:
(LASSO Regression, Variable Selection)

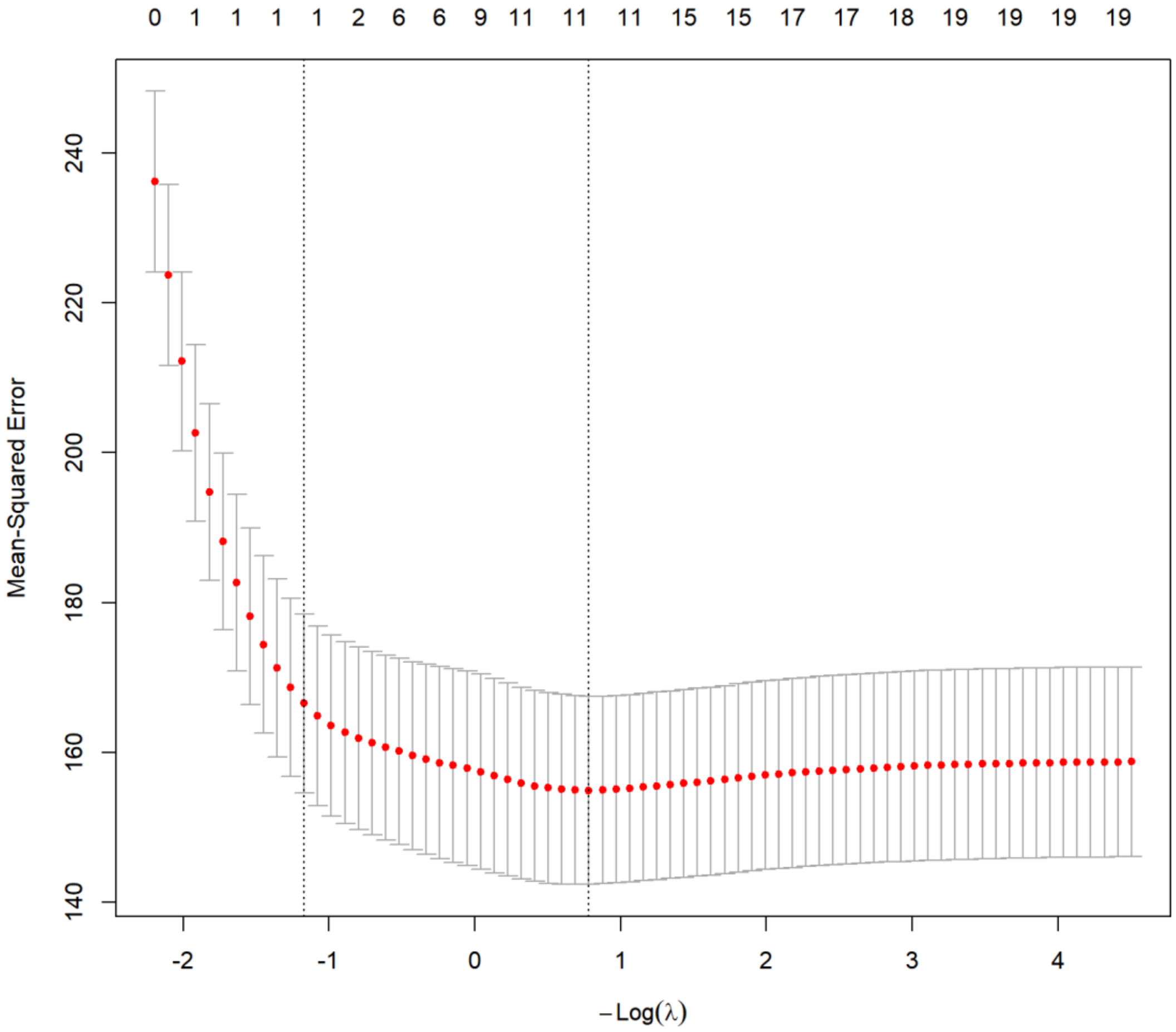

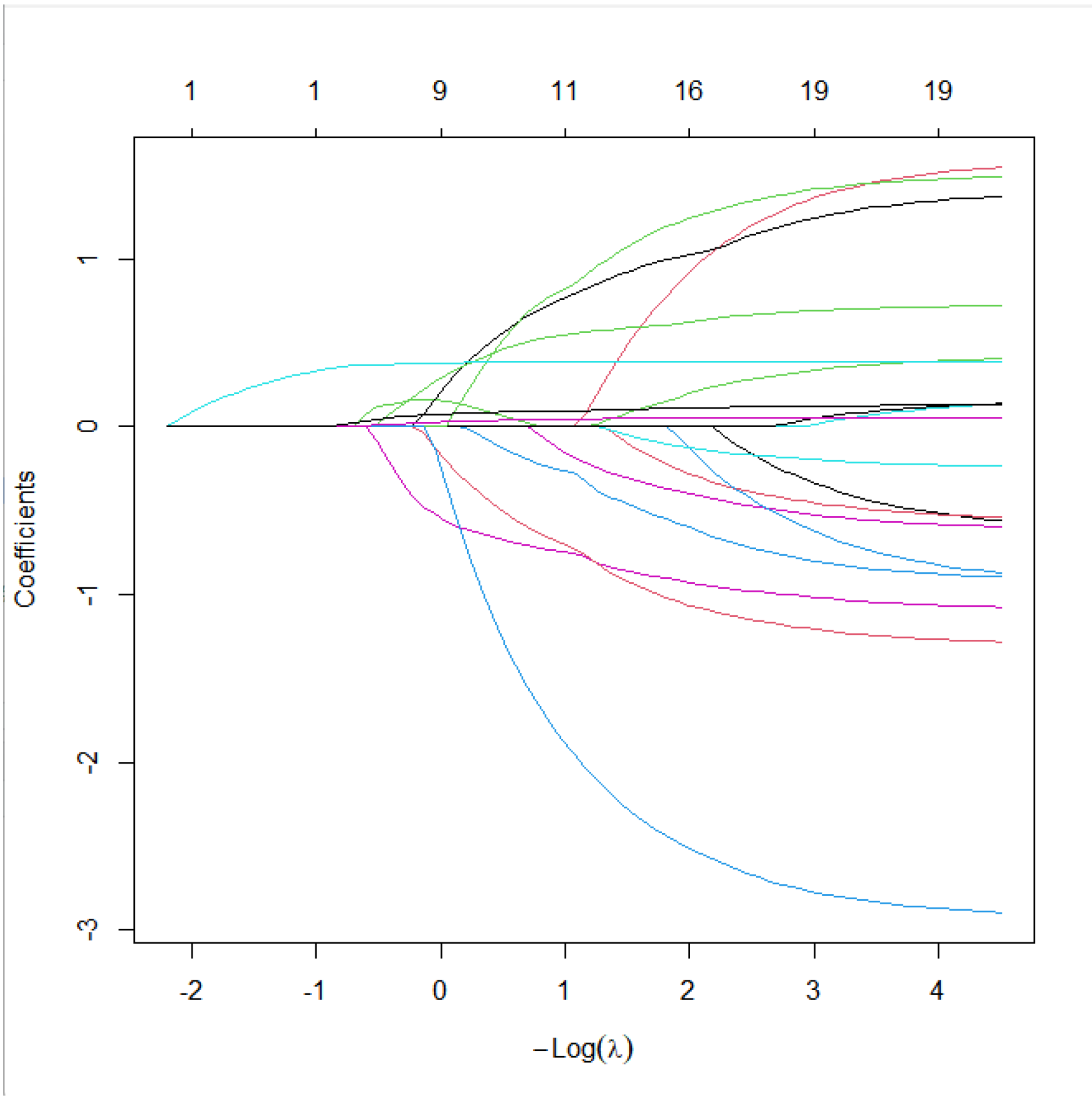

lambda.min 结果（最小 MSE）：

```
> print(results_min)
      Variable  Coefficient
1  (Intercept) 33.106532317
2           A3   0.005635037
3           A4  -0.224761332
4           A6  -0.043756971
5           A7   0.693821708
6           A9   0.518888164
7          A12  -0.722075914
8          A14  -0.632445332
9          A15   0.731865020
10         A16  -1.663715677
11         PAM   0.385158004
12      MAIA_2   0.041839246
```

```
13      Health  0.090459168
```

lambda.1se 结果（更简约模型）：

```
> print(results_1se)
     Variable Coefficient
1 (Intercept)  42.7406759
2         PAM   0.3049063
```
