# Supplementary material for: Patient activation and self-management in patients with coronary heart disease: the mediating role of eHealth literacy
Source: Front Public Health. 2026 Sep 11;14:1799712. doi: 10.3389/fpubh.2026.1799712 (PMC13612537; doi:10.3389/fpubh.2026.1799712)
Supplement: Supplementary file 2 [file Data_Sheet_2.pdf]

Supplement: LASOO

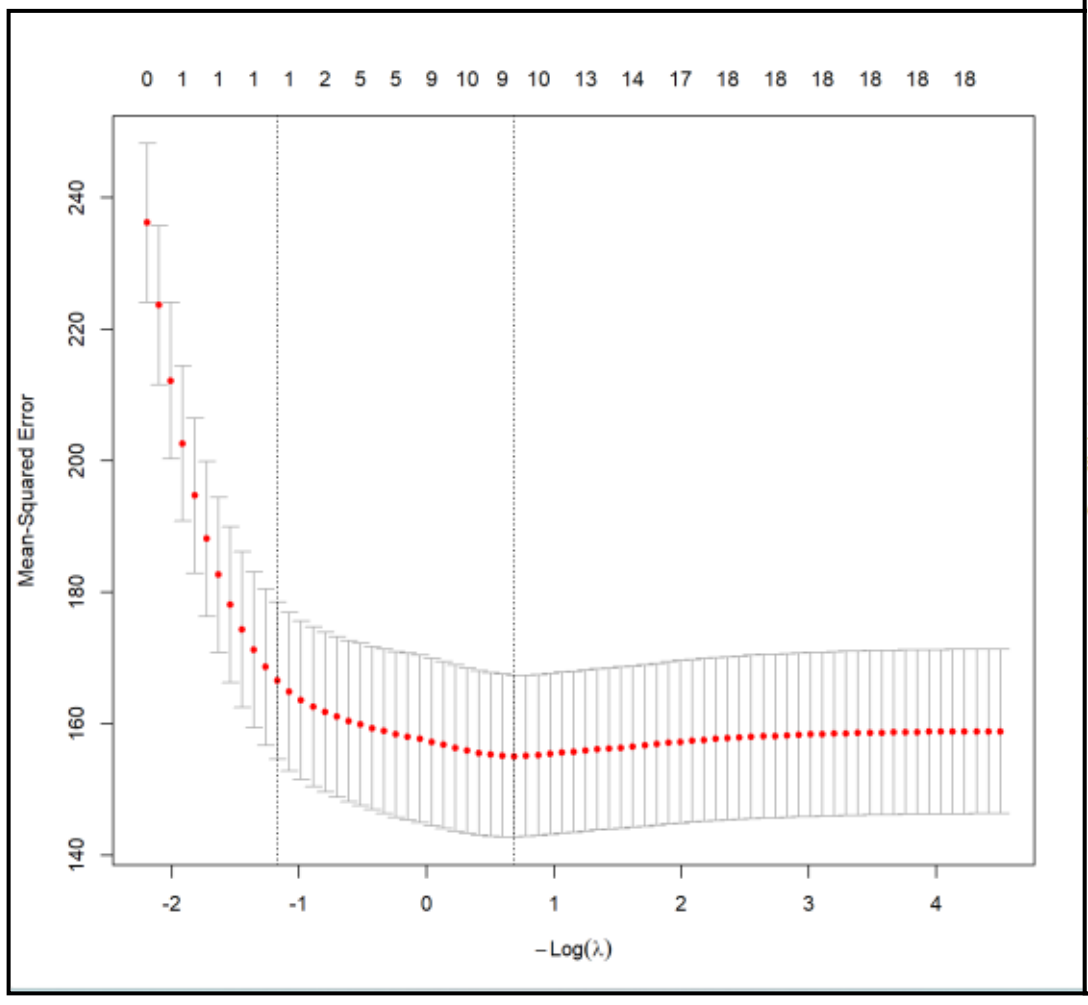

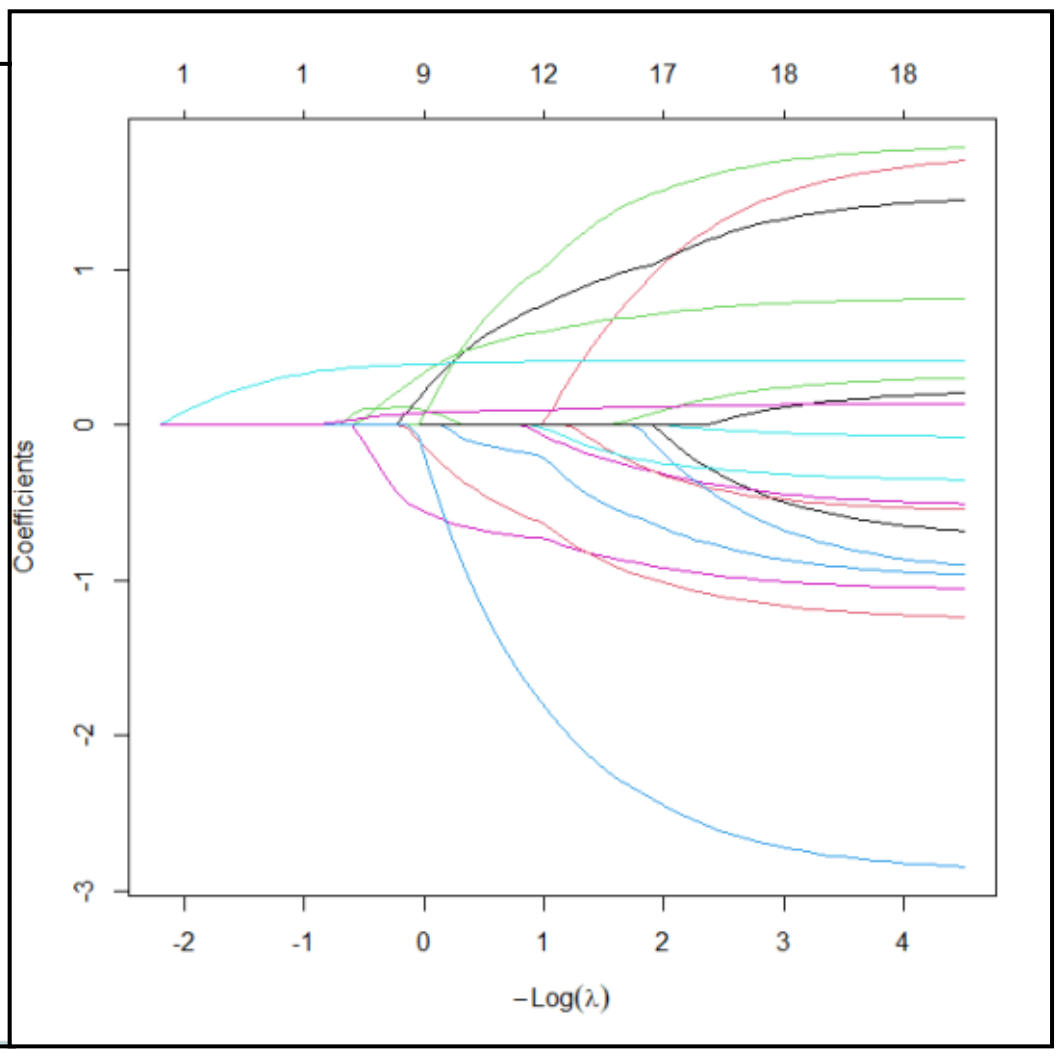

lambda.min 结果（最小 MSE）:> print(results_min)   Variable Coefficient

```
1  (Intercept) 35.30640834
2           A4 -0.15926063
3           A7  0.66264108
4           A9  0.55620790
5          A12 -0.70309821
6          A14 -0.53535785
7          A15  0.83681762
8          A16 -1.47489596
9          PAM  0.40532466
10      Health  0.09275523
```

lambda.1se 结果（更简约模型）:

```
> print(results_1se)
     Variable Coefficient
1 (Intercept)  42.7406759
2         PAM   0.3049063
```

从 lambda.1se 结果看得出来，没有协变量入选模型
