# Supplementary material for: Patient activation and self-management in patients with coronary heart disease: the mediating role of eHealth literacy
Source: Front Public Health. 2026 Sep 11;14:1799712. doi: 10.3389/fpubh.2026.1799712 (PMC13612537; doi:10.3389/fpubh.2026.1799712)
Supplement: Supplementary file 3 [file Table_1.doc]

**Supplementary table 1. Subgroup analysis**

| Subgroup | N | β (95%CI) | P for interaction |
| --- | --- | --- | --- |
| Age, n(%) |  |  | 0.188 |
| <60 years | 161 | 0.099(0.007,0.194) |  |
| ≥60 years | 272 | 0.017(-0.024,0.062) |  |
| Education, n(%) |  |  | 0.120 |
| ≤High school | 342 | 0.034(-0.007,0.079) |  |
| ≥College | 91 | 0.038(-0.071,0.147) |  |

**Age**

**Education**
