# Supplementary material for: Patient activation and self-management in patients with coronary heart disease: the mediating role of eHealth literacy
Source: Front Public Health. 2026 Sep 11;14:1799712. doi: 10.3389/fpubh.2026.1799712 (PMC13612537; doi:10.3389/fpubh.2026.1799712)
Supplement: Supplementary file 4 [file Table_2.doc]

**Supplementary table 2. mediation model with covariates**

**Total effect model：**

**lm(formula = HH_SESM ~ PAM + A1 + A2 + A3 + A4 + A5 + A6 + A7 +**

**A8 + A9 + A10 + A11 + A12 + A13 + A14 + A15 + A16, data = med_data)**

Residuals:

Min	1Q Median	3Q	Max

-37.219 -8.762 -0.063	8.180 34.554

Coefficients:

Estimate Standardized Std. Error t value Pr(>|t|)

(Intercept) 34.790502	NA	5.403881	6.438 3.55e-10 ***

PAM	0.433726	0.533419	0.035188 12.326 < 2e-16 ***

A12	-0.717068	-0.023309	1.672385 -0.429	0.6683

A22	5.962398	0.156558	2.567024	2.323	0.0207 *

A23	5.726769	0.182092	3.027121	1.892	0.0593 .

A24	5.967840	0.166355	3.257227	1.832	0.0677 .

A32	1.174929	0.035141	1.707081	0.688	0.4917

A33	0.999155	0.023139	2.393519	0.417	0.6766

A34	0.486485	0.008061	3.251637	0.150	0.8811

A35	3.114131	0.070693	3.338908	0.933	0.3516

A42	1.138748	0.035008	2.510351	0.454	0.6504

A43	-1.338843	-0.042571	2.434953 -0.550	0.5827

A52	-3.207140	-0.083853	3.217788 -0.997	0.3195

A53	-2.882410	-0.036528	4.395002 -0.656	0.5123

A54	-3.218269	-0.062805	3.744079 -0.860	0.3906

A62	0.313482	0.010119	2.879913	0.109	0.9134

A63	-0.979946	-0.029044	3.005675 -0.326	0.7446

A64	0.571768	0.010301	3.455892	0.165	0.8687

A72	-2.193694	-0.059681	1.878291 -1.168	0.2436

A73	2.275438	0.073836	2.006093	1.134	0.2574

A82	0.609170	0.019390	2.320512	0.263	0.7931

A83	1.022849	0.027725	2.400523	0.426	0.6703

A84	-5.182367	-0.057707	4.009488 -1.293	0.1969

A92	-1.325809	-0.031848	2.117453 -0.626	0.5316

A93	-0.729285	-0.020590	2.063616 -0.353	0.7240

A94	-1.030962	-0.022913	2.451836 -0.420	0.6744

A95	2.972642	0.081747	2.378319	1.250	0.2121

A102	-0.660200	-0.021325	1.508991 -0.438	0.6620

A112	0.967948	0.029705	1.529856	0.633	0.5273

A113	-2.096509	-0.042154	2.532110 -0.828	0.4082

A122	-0.659438	-0.020714	1.703568 -0.387	0.6989

A123	-2.496728	-0.065279	2.179113 -1.146	0.2526

A124	-0.288096	-0.004301	3.404088 -0.085	0.9326

A132	1.272002	0.025576	2.177679	0.584	0.5595

A133	-0.355961	-0.006163	2.429351 -0.147	0.8836

A134	0.518703	0.006190	3.383327	0.153	0.8782

A142	-2.422040	-0.063597	1.810768 -1.338	0.1818

A143	-3.738718	-0.085450	1.969852 -1.898	0.0584 .

A144	-3.478069	-0.041503	3.560842 -0.977	0.3293

A145	3.515904	0.024510	6.065129	0.580	0.5625

A152	2.607290	0.073151	1.592716	1.637	0.1024

A162	-3.022435	-0.092753	1.337712 -2.259	0.0244 *

---

Signif. codes: 0 ‘***’ 0.001 ‘**’ 0.01 ‘*’ 0.05 ‘.’ 0.1 ‘ ’ 1

Residual standard error: 12.31 on 391 degrees of freedom

Multiple R-squared: 0.4175,	Adjusted R-squared: 0.3564

F-statistic: 6.836 on 41 and 391 DF, p-value: < 2.2e-16

**# Mediating model**

**lm(formula = Health ~ PAM + A1 + A2 + A3 + A4 + A5 + A6 + A7 +**

**A8 + A9 + A10 + A11 + A12 + A13 + A14 + A15 + A16, data = med_data)**

Residuals:

Min	1Q	Median	3Q	Max

-19.3000 -4.1175	0.4569	4.7192 17.9948

Coefficients:

Estimate Standardized Std. Error t value Pr(>|t|)

(Intercept) 9.013967	NA	3.162765	2.850	0.0046 **

PAM	0.218071	0.460220	0.020595 10.589	<2e-16 ***

A12	1.299281	0.072475	0.978808	1.327	0.1851

A22	-1.236970	-0.055735	1.502419 -0.823	0.4108

A23	-4.264746	-0.232696	1.771703 -2.407	0.0165 *

A24	-4.619102	-0.220949	1.906379 -2.423	0.0158 *

A32	0.833081	0.042756	0.999114	0.834	0.4049

A33	2.326223	0.092443	1.400870	1.661	0.0976 .

A34	3.982799	0.113245	1.903107	2.093	0.0370 *

A35	1.099177	0.042818	1.954184	0.562	0.5741

A42	-0.671039	-0.035400	1.469249 -0.457	0.6481

A43	-0.452288	-0.024678	1.425121 -0.317	0.7511

A52	3.731985	0.167439	1.883296	1.982	0.0482 *

A53	1.509513	0.032827	2.572292	0.587	0.5577

A54	0.973651	0.032605	2.191322	0.444	0.6571

A62	0.718940	0.039822	1.685546	0.427	0.6700

A63	-0.888973	-0.045213	1.759151 -0.505	0.6136

A64	2.197001	0.067920	2.022652	1.086	0.2781

A72	-0.704217	-0.032876	1.099319 -0.641	0.5222

A73	-0.156325	-0.008705	1.174119 -0.133	0.8941

A82	-1.274354	-0.069607	1.358141 -0.938	0.3487

A83	-1.091823	-0.050783	1.404970 -0.777	0.4376

A84	-3.745477	-0.071569	2.346659 -1.596	0.1113

A92	1.370259	0.056484	1.239296	1.106	0.2695

A93	-0.187261	-0.009072	1.207786 -0.155	0.8769

A94	0.062476	0.002383	1.435002	0.044	0.9653

A95	1.068195	0.050407	1.391974	0.767	0.4433

A102	1.868770	0.103581	0.883177	2.116	0.0350 *

A112	0.386576	0.020357	0.895389	0.432	0.6662

A113	0.556633	0.019205	1.481985	0.376	0.7074

A122	1.720727	0.092753	0.997058	1.726	0.0852 .

A123	1.833407	0.082258	1.275384	1.438	0.1514

A124	4.317947	0.110628	1.992333	2.167	0.0308 *

A132	1.609777	0.055542	1.274544	1.263	0.2073

A133	-0.343513	-0.010206	1.421842 -0.242	0.8092

A134	-5.702365	-0.116764	1.980182 -2.880	0.0042 **

A142	0.296501	0.013360	1.059800	0.280	0.7798

A143	-0.356207	-0.013970	1.152908 -0.309	0.7575

A144	0.575633	0.011787	2.084078	0.276	0.7825

A145	-3.885063	-0.046475	3.549778 -1.094	0.2744

A152	0.554683	0.026705	0.932179	0.595	0.5522

A162	0.057606	0.003034	0.782931	0.074	0.9414

---

Signif. codes: 0 ‘***’ 0.001 ‘**’ 0.01 ‘*’ 0.05 ‘.’ 0.1 ‘ ’ 1

Residual standard error: 7.204 on 391 degrees of freedom

Multiple R-squared: 0.4125,	Adjusted R-squared: 0.3508

F-statistic: 6.695 on 41 and 391 DF, p-value: < 2.2e-16

**Direct effect model**

**lm(formula = HH_SESM ~ PAM + Health + A1 + A2 + A3 + A4 + A5 +**

**A6 + A7 + A8 + A9 + A10 + A11 + A12 + A13 + A14 + A15 + A16,**

**data = med_data)**

Residuals:

Min	1Q Median	3Q	Max

-36.818 -8.363 -0.154	8.035 35.971

Coefficients:

Estimate Standardized Std. Error t value Pr(>|t|)

(Intercept) 3.370e+01	NA 5.453e+00	6.181 1.61e-09 ***

PAM	4.074e-01	5.011e-01 3.987e-02 10.219 < 2e-16 ***

Health	1.207e-01	7.031e-02 8.630e-02	1.398	0.1629

A12	-8.738e-01	-2.841e-02 1.674e+00 -0.522	0.6020

A22	6.112e+00	1.605e-01 2.566e+00	2.382	0.0177 *

A23	6.241e+00	1.985e-01 3.046e+00	2.049	0.0411 *

A24	6.525e+00	1.819e-01 3.278e+00	1.991	0.0472 *

A32	1.074e+00	3.213e-02 1.707e+00	0.630	0.5293

A33	7.185e-01	1.664e-02 2.399e+00	0.299	0.7647

A34	5.938e-03	9.839e-05 3.266e+00	0.002	0.9986

A35	2.982e+00	6.768e-02 3.336e+00	0.894	0.3720

A42	1.220e+00	3.750e-02 2.508e+00	0.486	0.6270

A43	-1.284e+00	-4.084e-02 2.432e+00 -0.528	0.5978

A52	-3.657e+00	-9.563e-02 3.230e+00 -1.132	0.2582

A53	-3.065e+00	-3.884e-02 4.392e+00 -0.698	0.4857

A54	-3.336e+00	-6.510e-02 3.740e+00 -0.892	0.3730

A62	2.267e-01	7.319e-03 2.877e+00	0.079	0.9372
A63	-8.727e-01	-2.587e-02 3.003e+00 -0.291	0.7715

A64	3.067e-01	5.525e-03 3.457e+00	0.089	0.9294

A72	-2.109e+00	-5.737e-02 1.877e+00 -1.123	0.2619

A73	2.294e+00	7.445e-02 2.004e+00	1.145	0.2529

A82	7.629e-01	2.428e-02 2.320e+00	0.329	0.7425

A83	1.155e+00	3.130e-02 2.399e+00	0.481	0.6307

A84	-4.730e+00	-5.267e-02 4.018e+00 -1.177	0.2397

A92	-1.491e+00	-3.582e-02 2.118e+00 -0.704	0.4819

A93	-7.067e-01	-1.995e-02 2.061e+00 -0.343	0.7319

A94	-1.038e+00	-2.308e-02 2.449e+00 -0.424	0.6717

A95	2.844e+00	7.820e-02 2.377e+00	1.196	0.2323

A102	-8.857e-01	-2.861e-02 1.516e+00 -0.584	0.5593

A112	9.213e-01	2.827e-02 1.528e+00	0.603	0.5470

A113	-2.164e+00	-4.350e-02 2.529e+00 -0.855	0.3929

A122	-8.671e-01	-2.724e-02 1.708e+00 -0.508	0.6120

A123	-2.718e+00	-7.106e-02 2.182e+00 -1.246	0.2137

A124	-8.091e-01	-1.208e-02 3.420e+00 -0.237	0.8131

A132	1.078e+00	2.167e-02 2.179e+00	0.495	0.6212

A133	-3.145e-01	-5.445e-03 2.427e+00 -0.130	0.8969

A134	1.207e+00	1.440e-02 3.415e+00	0.353	0.7240

A142	-2.458e+00	-6.454e-02 1.809e+00 -1.359	0.1750

A143	-3.696e+00	-8.447e-02 1.968e+00 -1.878	0.0611 .

A144	-3.548e+00	-4.233e-02 3.557e+00 -0.997	0.3192

A145	3.985e+00	2.778e-02 6.067e+00	0.657	0.5117

A152	2.540e+00	7.127e-02 1.591e+00	1.596	0.1113

A162	-3.029e+00	-9.297e-02 1.336e+00 -2.267	0.0239 *

---

Signif. codes: 0 ‘***’ 0.001 ‘**’ 0.01 ‘*’ 0.05 ‘.’ 0.1 ‘ ’ 1

Residual standard error: 12.29 on 390 degrees of freedom

Multiple R-squared: 0.4204,	Adjusted R-squared: 0.358

F-statistic: 6.736 on 42 and 390 DF, p-value: < 2.2e-16
