# Supplementary material for: Patient activation and self-management in patients with coronary heart disease: the mediating role of eHealth literacy
Source: Front Public Health. 2026 Sep 11;14:1799712. doi: 10.3389/fpubh.2026.1799712 (PMC13612537; doi:10.3389/fpubh.2026.1799712)
Supplement: Supplementary file 5 [file Table_3.doc]

**Supplementary table 3. common method bias**

Call: fa(r = cmv_vars, nfactors = 1, rotate = "none", fm = "pa")

Standardized loadings (pattern matrix) based upon correlation matrix

PA1	h2	u2 com

PAM	0.89 0.79 0.21	1

Health 0.57 0.33 0.67	1

HH_SESM 0.66 0.43 0.57	1

PA1

SS loadings	1.55

Proportion Var 0.52

Mean item complexity = 1

Test of the hypothesis that 1 factor is sufficient.

df null model = 3 with the objective function = 0.73 with Chi Square = 314.1

df of the model are 0 and the objective function was 0

The root mean square of the residuals (RMSR) is 0

The df corrected root mean square of the residuals is NA

The harmonic n.obs is 433 with the empirical chi square 0.01 with prob < NA

The total n.obs was 433 with Likelihood Chi Square = 0.02 with prob < NA

Tucker Lewis Index of factoring reliability = -Inf

Fit based upon off diagonal values = 1

Measures of factor score adequacy

PA1

Correlation of (regression) scores with factors	0.91

Multiple R square of scores with factors	0.83

Minimum correlation of possible factor scores	0.66
